# Impact of 2′-Fucosyllactose on Gut Microbiota Composition in Adults with Chronic Gastrointestinal Conditions: Batch Culture Fermentation Model and Pilot Clinical Trial Findings

**DOI:** 10.3390/nu13030938

**Published:** 2021-03-14

**Authors:** Jennifer Joan Ryan, Andrea Monteagudo-Mera, Nikhat Contractor, Glenn R. Gibson

**Affiliations:** 1 Helfgott Research Institute, National University of Natural Medicine, Portland, OR 97201, USA; 2 Department of Food and Nutritional Sciences, University of Reading, Reading RG6 6AD, UK; a.monteagudo@reading.ac.uk (A.M.-M.); g.r.gibson@reading.ac.uk (G.R.G.); 3 Metagenics, Inc., Aliso Viejo, CA 92656, USA; nikkycontractor@metagenics.com

**Keywords:** 2′-FL, dysbiosis, fucosyllactose, gut microbiota, human milk oligosaccharides, irritable bowel syndrome, inflammatory bowel disease, prebiotics, probiotics

## Abstract

Intestinal dysbiosis has been described in patients with certain gastrointestinal conditions including irritable bowel syndrome (IBS) and ulcerative colitis. 2′-fucosyllactose (2′-FL), a prebiotic human milk oligosaccharide, is considered bifidogenic and butyrogenic. To assess prebiotic effects of 2′-FL, alone or in combination with probiotic strains (potential synbiotics), *in vitro* experiments were conducted on stool from healthy, IBS, and ulcerative colitis adult donors. In anaerobic batch culture fermenters, *Bifidobacterium* and *Eubacterium rectale*-*Clostridium coccoides* counts, and short-chain fatty acids (SCFAs) including butyrate increased during fermentation with 2′-FL and some of the 2′-FL/probiotic combinations. In a subsequent open-label pilot trial, the effect of a 2′-FL-containing nutritional formula was evaluated in twelve adults with IBS or ulcerative colitis. Gastrointestinal Quality of Life Index (GIQLI) total and gastrointestinal symptoms domain scores, stool counts of *Bifidobacterium* and *Faecalibacterium prausnitzii*, and stool SCFAs including butyrate, increased after six weeks of intervention. Consistent with documented effects of 2′-FL, the batch culture fermentation experiments demonstrated bifidogenic and butyrogenic effects of 2′-FL during fermentation with human stool samples. Consumption of the 2′-FL-containing nutritional formula by adults with IBS or ulcerative colitis was associated with improvements in intra- and extra-intestinal symptoms, and bifidogenic and butyrogenic effects.

## 1. Introduction

Irritable bowel syndrome (IBS) [1], inflammatory bowel disease (IBD) [2], and celiac disease [3] are chronic gastrointestinal conditions with predominantly differing pathogenesis. Interestingly, these conditions are characterized by at least some level of chronic intestinal mucosal inflammation [4,5,6]. However, in contrast to patients with IBD and celiac disease, the intestinal inflammation in patients with IBS is considered low grade [4,5].

Pathophysiology shared among individuals with IBS, IBD, and celiac disease includes dysbiosis, defined as “any change to the composition of resident commensal communities relative to the community found in healthy individuals” [7]. Patients with IBS [8,9], IBD [10,11], and celiac disease [12] may have reduced stool counts of the genus *Bifidobacterium* and the butyrate-producing species *Faecalibacterium prausnitzii*.

According to recent consensus statements by the International Scientific Association for Probiotics and Prebiotics (ISAPP), a prebiotic is defined as “a substrate that is selectively utilized by host microorganisms conferring a health benefit” [13], probiotics are defined as “live microorganisms that, when administered in adequate amounts, confer a health benefit on the host” [14], and a synbiotic is defined as “a mixture comprising live microorganisms and substrate(s) selectively utilized by host microorganisms that confers a health benefit on the host” [15]. Prebiotics and probiotics represent non-pharmacologic approaches for addressing dysbiosis associated with IBS [16,17,18], IBD [19,20], and celiac disease [21]. Prebiotics support the growth and activity of specific health-promoting, non-pathogenic microorganisms such as *Bifidobacterium* [13]. Probiotic mechanisms among a diversity of strains include normalization of perturbed microbiota, regulation of intestinal transit, and short-chain fatty acid (SCFA) production [14]. SCFAs are the main end products of anaerobic microbial fermentation in the mammalian colon [22,23]. Rapidly absorbed by the intestinal epithelium, the pleiotropic SCFA butyrate modulates visceral sensitivity [24] and intestinal motility [24], and regulates intestinal inflammation through multiple mechanisms including inhibition of nuclear factor-kappa B (NFkB) activation [24,25].

Dietary prebiotics can stimulate endogenous intestinal bacteria, having the effect of promoting butyrate production [22,26,27]. Due to abilities to increase the activity and growth of bifidobacteria and butyrate-producers via cross-feeding mechanisms, specific prebiotics have been described as having both bifidogenic and butyrogenic effects [26,28,29]. 2′-fucosyllactose (2′-FL) is a trisaccharide prebiotic and is the human milk oligosaccharide (HMO) most abundantly produced by the majority of nursing mothers [30]. However, whether or not a nursing mother produces 2′-FL depends on whether or not they are secretors of the fucosyltransferase 2 (FUT2) gene [31]. Serving as growth substrates for intestinal bacteria in the distal colon, HMOs act as the first prebiotics consumed by nursing infants [32,33,34,35]. In the human gastrointestinal tract, *Bifidobacterium* species are the main utilizers of HMOs, which are metabolized to the SCFA acetate and lactic acid [35,36].

Health benefits to infants are apparent for HMOs [37,38] including for supplementation of 2′-FL given to formula-fed infants [39,40]. Supplemental 2′-FL and lactose-N-neotetraose, synthesized and structurally similar to that found in human milk, has been associated with a bifidogenic effect in infants [40]. However, data on potential benefits of supplemental 2′-FL in adult populations are extremely limited. Elison et al. [41] reported that oral supplementation with 2′-FL resulted in increased stool counts of *Bifidobacterium* in healthy adults and Iribarren et al. [42] recently reported that a combination of 2′-FL and lactose-N-neotetraose resulted in increased stool counts of *Bifidobacterium*.

Given that patients with specific chronic gastrointestinal conditions exhibit dysbiosis (including reduced *Bifidobacterium*) and 2′-FL may induce bifidogenic effects, anaerobic batch culture experiments were implemented to assess prebiotic effects of 2′-FL. Alone or in combination with different probiotic strains (potential synbiotics), impact of 2′-FL on the composition and metabolic activity of stool microbiota from healthy adult volunteers and individuals with IBS or ulcerative colitis was evaluated. Several strain-identified, commercially-available probiotic species selected from the most commonly used and clinically-recommended bacterial genera of probiotics, *Lactobacillus* and *Bifidobacterium* [43], were tested. A subsequent pilot trial was conducted to examine the impact of a 2′-FL-containing nutritional formula on gastrointestinal quality of life, stool counts of commensal microbiota, and stool SCFA concentrations in adults with IBS, IBD and celiac disease.

## 2. Materials and Methods

### 2.1. In Vitro Experiments

#### 2.1.1. 2′-FL and Probiotic Strains

Five potential synbiotic combinations were tested as described in Table 1. The 2′-FL and probiotic strains were supplied by Metagenics, Inc. (Aliso Viejo, CA, USA). Probiotic suspensions were prepared for addition to fermenter vessels by centrifuging at 14,000× *g* for 5 min. The supernatant was removed and cells were washed twice in anaerobic phosphate buffer (1 M, pH 7.4). Cells were re-suspended in 1 mL phosphate buffer at a concentration of 10^7^ CFU/mL immediately before addition to fermenter vessels.

#### 2.1.2. Batch Cultures

Anaerobic batch culture experiments were used to investigate the ability of stool microbiota to utilize 2′-FL and to test different potential synbiotic combinations containing 2′-FL. These were performed in three populations in triplicate using stool samples from 9 different volunteers (healthy, n = 3; IBS, n = 3; ulcerative colitis, n = 3). Donors were aged between 24 and 40 years old, who had not received antibiotics, or prebiotic or probiotics supplements for at least six months prior to sample collection. IBS donors had received a medical diagnosis and were not under any treatment at the time of the experiment. Ulcerative colitis donors were being treated with mesalazine, and all were in remission at the time of this study. Sterile gently stirred pH-controlled batch culture 10 mL fermentation vessels were aseptically filled with 9 mL of sterile (autoclaved) basal nutrient medium and sparged with O_2_-free N_2_ (15 mL/min) overnight to establish anaerobic conditions. The basal medium (per liter) consisted of: 2 g peptone water, 2 g yeast extract, 0.1 g NaCl, 0.04 g K_2_HPO_4_, 0.04 g KH_2_PO_4_, 0.01 g MgSO_4_.7H_2_O, 0.01 g CaCl.6H_2_O, 2 g NaHCO_3_, 2 mL Tween 80, 0.05 g hemin, 0.01 mL vitamin K1, 0.5 g L-cysteine-HCl, 0.5 g bile salt and 4 mL resazurin solution (0.25 g/L). 2′-FL (1% *w*/*v*) and probiotic combinations (10^7^ CFU per strain) were added to fermentation vessels just before the addition of the fecal slurry which was prepared by homogenizing fresh human feces (10%, *w*/*v*) in anaerobic phosphate-buffered saline. Vessels were kept at 37 °C using a circulating water bath and pH controlled between 6.7 and 6.9 using an automated pH controller (Fermac 260, Electrolab, Tewkesbury, UK). Each vessel was inoculated with 1 mL of fresh fecal slurry. Additionally, vessels with fructooligosaccharides (FOS)/inulin mixture (1% *w*/*v*) and without any substrate were used as positive and negative controls respectively. Batch culture fermentations were run for 24 h and samples were collected at 0, 8, and 24 h.

#### 2.1.3. Flow Cytometry-Fluorescence In Situ Hybridization for Bacterial Enumerations

Samples (750 µL) were centrifuged at 13,000× *g* for 5 min at room temperature. Supernatants were collected and filtered through a 0.22 µm Millipore syringe filter for gas chromatography analysis. Pellets were fixed for further fluorescence in situ hybridization and kept at −20 °C. Briefly, after centrifugation pellets were resuspended in 375 µL of 1X PBS and 1150 µL of cold 4% (*v*/*v*) paraformaldehyde. The suspension was mixed and stored at 4 °C for 4–6 h. After incubation, samples were washed twice with 1 mL of 1X PBS. Finally, samples were centrifuged at 13,000× *g* for 5 min, supernatant was discarded and the pellet was resuspended in 300 µl of 1X PBS and 300 µL of ethanol. Samples were vortexed and stored at −20 °C for further analysis.

For Flow-FISH cytometry, we used 75 µL of the fixed samples. Fixed cells were washed twice with PBS and pre-treated for 10 min with lysozyme at 1 mg/mL. Cells were resuspended in 1 mL hybridization buffer. All hybridizations were performed in the dark at 35 °C overnight in the hybridization solution containing genus- and group-specific 16S rRNA-targeted oligonucleotide probes. Cells were centrifuged at 10,000× *g* for 3 min, resuspended in pre-warmed washing buffer and incubated at 37 °C for 20 min to remove non-specific binding of the probe. Finally, cells were centrifuged at 10,000× *g* for 3 min and resuspended in PBS for flow cytometry analysis. Probes used are described in Table 2.

**Table 2:** 16S rRNA-Targeted Oligonucleotide Probes Used for *In Vitro* Experiments.

Probe Name	Sequence (5′ to 3′)	Targeted Bacterial Group	Reference
Non Eub	ACTCCTACGGGAGGCAGC	Negative control	Wallner et al., 1993 [44]
Eub338	GCTGCCTCCCGTAGGAGT	Total bacteria	Daims et al., 1999 [45]
Eub338II	GCAGCCACCCGTAGGTGT	Total bacteria	Daims et al., 1999 [45]
Eub338III	GCTGCCACCCGTAGGTGT	Total bacteria	Daims et al., 1999 [45]
Ato291	GGTCGGTCTCTCAACCC	*Atopobium* cluster	Harmsen et al., 2000 [46]
Bac303	CCAATGTGGGGGACCTT	*Bacteroides*/*Prevotella*	Manz et al., 1996 [47]
Bif164	CATCCGGCATTACCACCC	*Bifidobacterium* spp.	Langendijk et al., 1995 [48]
Chis150	TTATGCGGTATTAATCTYCCTTT	*Clostridium histolyticum* (*Clostridium* cluster I and II)	Franks et al., 1998 [49]
Prop853	ATTGCGTTAACTCCGGCAC	*Clostridium* cluster IX	Walker et al., 2005 [50]
DSV687	TACGGATTTCACTCCT	*Desulfovibrio* spp.	Hold et al., 2003 [51]
Erec482	GCTTCTTAGTCARGTACCG	*Eubacterium rectale*/*Clostridium coccoides* (*Clostridium* cluster IVXa and IVXb)	Franks et al., 1998 [49]
Fprau655	CGCCTACCTCTGCACTAC	*Faecalibacterium prausnitzii*	Devereux et al., 1992 [52]
Lab158	GGTATTAGCAYCTGTTTCCA	*Lactobacillus*/*Enterococcus*	Harmsen et al., 1999 [53]
Rrec584	TCAGACTTGCCGYACCGC	*Roseburia* spp.	Walker et al., 2005 [50]

#### 2.1.4. *In Vitro* SCFA Analysis

Samples from each fermentation time were centrifuged at 13,000× *g* for 10 min to obtain the supernatants which were kept at −20 °C until analysis. Before analysis, samples were extracted and derivatised following the method described by Ferreira-Lazarte et al. [54]. A 5890 Series II Gas Chromatograph (Hewlett Packard) fitted with a Rtx-1 10 m × 0.18 mm column with a 0.20 μm coating (Crossbond 100% dimethyl polysiloxane; Restek) was used for analysis. Helium was used as the carrier gas at a flow rate of 0.7 mL/min. Injector and detector temperatures were 275 °C. Oven temperature was programmed from 63 °C for 3 min and then heated to 190 °C at a heating rate of 3 °C/min and held at 190 °C for 3 min. SCFA standards analysis was also carried out to quantify concentrations of all compounds.

#### 2.1.5. Statistical Analysis of *In Vitro* Data

Statistical analyses were performed using Graph Pad Prism 8 (Graph Pad Software Inc., San Diego, CA, USA). One-way ANOVA with Dunnet’s multiple comparison test was used to determine significant differences from baseline within the same substrate. Differences were considered to be significant when *p* < 0.05.

### 2.2. Pilot Clinical Trial

#### 2.2.1. Clinical Trial Design

To evaluate potential effects of a 2′-FL-containing nutritional formula, a single-arm, open-label, pilot trial was implemented. The clinical trial protocol was approved by Quorum Review Board (Seattle, WA, USA) and registered at ClincalTrials.gov (NCT03011593). Participants provided written informed consent prior to participation in this study.

The interventional period included two clinical visits with questionnaires administered and stool samples collected at baseline and at the study end point (after six weeks). The Gastrointestinal Quality of Life Index (GIQLI) [55], administered to all participants, was the primary outcome measure. Secondary outcome measures were the Digestive Symptom Frequency Questionnaire (DSFQ) [56] and the Inflammatory Bowel Disease Questionnaire (IBDQ) [57], administered only to participants with IBS and IBD, respectively. Exploratory outcome measures were stool CFU counts of commensal bacteria and stool SCFA levels.

#### 2.2.2. Clinical Trial Participants and Recruitment

Up to twenty adults aged 21–75 years, with a body mass index (BMI) of 19–40 kg/m^2^, and with previously diagnosed IBS, ulcerative colitis, Crohn’s disease or celiac disease were recruited at four medical clinics in the United States (located in La Jolla, CA; Forney, TX; Avon, CT; and South Orange, NJ). Potential participants were approached by clinicians associated with this study. Target enrollment was up to twenty individuals. Exclusion criteria were as follows: taking oral or intravenous antibiotic, antiparasitic, or antifungal medications; gastrointestinal surgery within three months prior to screening; currently having a colostomy or ileostomy bag in place; cancer within the last five years; or women who were lactating, pregnant or planning pregnancy during the study period. Furthermore, participants were excluded if they had done any of the following within 28 days prior to screening: initiated or made changes to medications or supplements, initiated or made changes to an exercise regimen or food plan, participated in a significant diet or weight loss program, used recreational drugs/substances, or participated in another research study. Unless medically indicated, participants were asked to refrain from making changes to their medications or supplements, exercise routine or diet for the duration of their participation in this study.

#### 2.2.3. Clinical Trial Intervention

The studied nutritional formula was manufactured and supplied by Metagenics, Inc. (Aliso Viejo, CA, USA) in fourteen-serving containers. The nutritional formula contained two grams per serving of 2′-FL plus micronutrients (vitamins A, B_6_, B_12_, C, D_3_, and E, biotin, calcium, chromium, copper, folate, iodine, iron, magnesium, manganese, pantothenic acid, phosphorus, riboflavin, selenium, thiamin, and zinc), macronutrients (protein, carbohydrates, fat, and fiber), amino acids, and isomalto-oligosaccharide. Participants were asked to add two scoops of the nutritional formula (40 g) to chilled water or juice (8–10 ounces) and drink it as a reconstituted beverage for six weeks. Participants consumed the beverage twice per day and therefore were administered four grams 2′-FL per day.

#### 2.2.4. Quality of Life Assessment and Stool Sample Analysis

The GIQLI, DSFQ, and IBDQ were scored as previously described [55,56,57]. Stool samples were shipped to Genova Diagnostics (Asheville, NC, USA) within twenty-four hours of collection; commensal gut microbiota were identified using 16S ribosomal RNA gene PCR and concentrations of SCFA were measured using gas chromatography–mass spectrometry (GC-MS) as previously described [58].

#### 2.2.5. Statistical Analysis of Clinical Trial Data

Given that the clinical trial was a pilot trial with data collected at only two time points, statistical analyses were limited to data from participants who completed the trial. Study measures are presented as the mean and standard deviation (SD) at each time point. For questionnaire and SCFA data, changes from baseline to study completion were analyzed using paired *t*-tests to identify significant differences. Microbiota PCR data were log transformed prior to calculation of geometric mean percent change and analyzed using paired *t*-tests. Statistically-significant (*p* < 0.05) microbiota parameters were subsequently evaluated using a Wilcoxon signed rank analysis, a more conservative test, to confirm statistical significance. In the case of microbiota PCR data that were outside of laboratory detection limits, the extreme detectable value was imputed for analysis. Given the small sample size of this study, effect size as Cohen’s d was calculated between baseline and the study end point for all continuous measures. Cohen’s d was calculated as (week 6 mean–baseline mean)/(baseline standard deviation) and interpreted as small (d = 0.20), medium (d = 0.50), or large (d = 0.80) effect size. Statistical analyses were performed using R (The R Foundation for Statistical Computing, software version 3.6.0, Vienna, Austria).

## 3. Results

### 3.1. In Vitro Study

#### 3.1.1. Effect of Fermentation on Bacterial Populations

**Figure 1:**
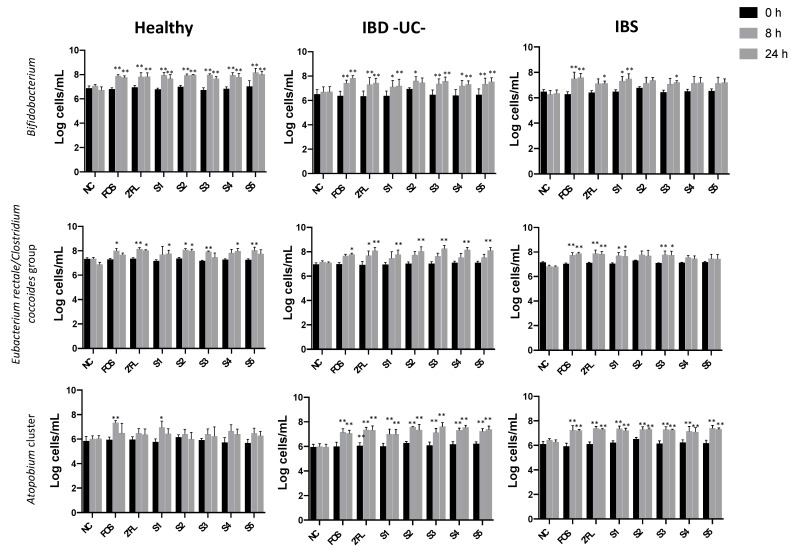
*Bifidobacterium*, *Clostridium coccoides*/*Eubacterium rectale* and *Atopobium* cluster populations at 0, 8, and 24 h of fermentation with no substrate/negative control (NC); positive control (FOS/inulin mixture); 2′-FL; S1: 2′-FL + *L. acidophilus* NCFM + *B. lactis* Bi-07; S2: 2′-FL+ *L. acidophilus* NCFM + *B. lactis* Bi-07 + *B. lactis* Bl-04 + *L. paracasei* Lpc-37; S3: 2′-FL + *L. acidophilus* NCFM + *B. lactis* Bi-07 + *L. rhamnosus* GG; S4: 2′-FL + *L. rhamnosus* GG; S5: 2′-FL + *L. salivarius* UCC118. Error bars show SEM (n = 3). Significant differences from baseline (time 0) are denoted with * (*p* ≤ 0.05) or ** (*p* ≤ 0.01).

Bacterial counts obtained by Flow-FISH are shown in Supplementary Figure S1 (Figure S1). The most noticeable change in the microbiota composition along the fermentations of stool samples from all groups was a significant increase in *Bifidobacterium* at 8 and 24 h of fermentation with the positive control, the 2′-FL, and several 2′-FL/probiotic combinations compared to baseline (Figure 1). Although the observed bifidogenic effect was significant overall, the increment was smaller with IBS donors due to higher inter-individual variation in the IBS donors. Closer examination, donor by donor in the IBS group, showed that this variation was due a low bifidogenic response (0.5 log) to 2′-FL and 2′-FL/probiotic combinations in one of the three IBS donors. However, the bifidogenic effect observed in the two additional IBS donors was similar to fermentations with healthy and ulcerative colitis donors. In addition, abundance of *Eubacterium rectale*-*Clostridium coccoides* (butyrate producers) was also significantly higher in presence of 2′-FL and 2′-FL/probiotic combinations after 8 h of fermentation (Figure 1). *Roseburia* species, another butyrate producer, also displayed a significant increment at 8 h in response of FOS/inulin mixture and 2′-FL in healthy and IBS fermentations but not in ulcerative colitis fermentations (Supplementary Figure S1). Interestingly, unlike healthy donors, stool microbiota of IBS and ulcerative colitis donors displayed a significant increase in *Atopobium* in response to 2′-FL and potential synbiotic treatments (Figure 1). Overall, no discernible differences were observed when 2′-FL/probiotic combinations were compared to 2′-FL alone (Figure 1).

#### 3.1.2. Effect of Fermentation on Short-Chain Fatty Acid (SCFA) Production

**Figure 2:**
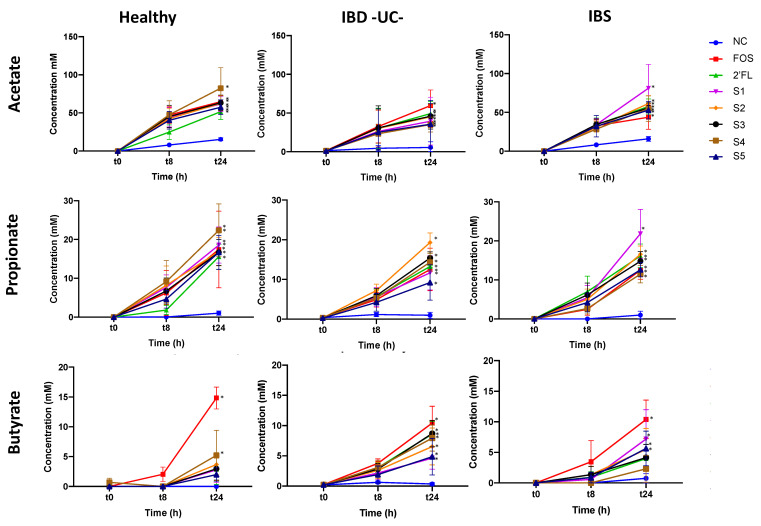
Acetate, propionate and butyrate concentration at 0, 8, and 24 h of fermentation with no substrate/negative control (NC); positive control (FOS/inulin mixture); 2′-FL; S1: 2′-FL + *L. acidophilus* NCFM + *B. lactis* Bi-07; S2: 2′-FL + *L. acidophilus* NCFM + *B. lactis* Bi-07 + *B. lactis* Bl-04 + *L. paracasei* Lpc-37; S3: 2′-FL + *L. acidophilus* NCFM + *B. lactis* Bi07 + *L. rhamnosus* GG; S4: 2′-FL + *L. rhamnosus* GG; S5: 2′-FL + *L. salivarius* UCC118. Error bars show SEM (n = 3). Significant differences from baseline (time 0) are denoted with * (*p* ≤ 0.05) or ** (*p* ≤ 0.01).

Acetate was the main SCFA detected during the fermentation of all prebiotics and potential synbiotics tested and its production accounted for approximately 75% of total SCFAs. Significant increments of acetate and propionate (the second most prevalent SCFA) were observed at the end of fermentation for all substrates and the three donor groups (Figure 2). Moderate increments of butyrate were also observed after 24 h. Although this increment was only significant for FOS and one 2′-FL/probiotic combination in fermentations with stool microbiota of healthy donors, significant increases in butyrate with 2′-FL and 2′-FL/probiotic combinations were detected in ulcerative colitis donor fermentations and also with FOS and some 2′-FL/probiotic combinations in IBS donor fermentations (Figure 2).

### 3.2. Pilot Clinical Trial

#### 3.2.1. Participant Characteristics

Twenty participants enrolled in this study and twelve completed it. Demographic parameters of the twelve participants who completed this study are presented in Table 3. The mean BMI of these twelve individuals was 23.8 kg/m^2^ (with a range from 19.4 to 29.6 kg/m^2^). The eight individuals who did not complete this study included two that dropped out/declined to participate before beginning the study intervention, three that dropped out due to non-serious adverse events, and three who were lost to follow up. The participants who dropped out due to adverse events reported worsening of pre-existing gastrointestinal symptoms, gastrointestinal upset, and a non-study-related viral infection. Data from the twelve participants that completed this study were analyzed.

#### 3.2.2. Impact on Gastrointestinal Quality of Life

Compared to baseline, some gastrointestinal quality of life questionnaire scores improved after six weeks (Table 4). Gastrointestinal Quality of Life Index (GIQLI) (administered to all participants) total score and “gastrointestinal symptoms” domain score increased (*p* < 0.05) with a medium effect size (d = 0.59 and d = 0.79, respectively). GIQLI “Social function” domain score increased (*p* < 0.01) with a small effect size (d = 0.42). Inflammatory Bowel Disease Questionnaire (IBDQ) (administered only to participants with IBD) domain score increased (*p* < 0.001) compared to baseline, with a large effect size (d = 1.65). Digestive Symptom Frequency Questionnaire (DSFQ) (administered only to participants with IBS) score did not change.

#### 3.2.3. Impact on Stool Bacteria Counts

In the clinical trial, counts of the majority of analyzed stool bacteria did not change after consuming the 2′-FL-containing nutritional formula for six weeks (Table 5). However, increases were observed in the following: *Bifidobacterium* species, *Bifidobacterium longum*, *Faecalibacterium prausnitzii*, *Anaerotruncus colihominis*, and *Pseudoflavonifractor* species (*p* < 0.05) with medium or greater effect sizes (d > 0.5).

#### 3.2.4. Impact on Stool Short-Chain Fatty Acids (SCFAs)

After six weeks, butyrate, acetate, and total stool SCFAs increased (*p* < 0.05) with large effect sizes (d ≥ 0.80) (Table 6).

## 4. Discussion

In the batch culture fermentation model, 2′-FL demonstrated prebiotic effects with increases in *Bifidobacterium* and SCFAs as well as substantial increments in other beneficial groups of the microbiota as *Clostridium* cluster XIVa and *Roseburia* spp. (butyrate producers). A previous *in vitro* study has also shown the ability of *Bifidobacterium* and *Eubacterium* to utilize fucosyllactose [29].

During fermentation, 2′-FL showed a bifidogenic effect similar to the positive control FOS/inulin mixture. The impact of 2′-FL on *Bifidobacterium* after 24 h was similar in the microbiota of all donors except for one individual with IBS. In the case of stool from this volunteer, no bifidogenic effect was detected with the positive control either, although *Roseburia spp.* increased with the treatments. Interestingly, this donor showed a two-fold abundance of *Roseburia spp.* over *Bifidobacterium* at baseline. This numerical superiority possibly provided an important competitive advantage for substrate consumption. Overall, *Bifidobacterium* and *Eubacterium rectale*-*Clostridium coccoides* were the main groups affected by 2′-FL and 2′-FL/probiotic combinations in fermentations of samples from the three donor groups.

Interestingly, the abundance of *Atopobium* cluster was also significantly increased in the presence of prebiotics and potential synbiotic combinations in individuals with IBS and ulcerative colitis. Slight, non-significant, increments were detected in this bacterial group in healthy donors. The role of *Atopobium* cluster in the human colon is not clear. However, they are commonly isolated from healthy human stool and are typically present at 1.5–3% of the total stool population. *Atopobium* cluster comprises most *Coriobacteriaceae* species, including the *Coriobacterium* group [47].

Using the Simulator of the Human Intestinal Microbial Ecosystem (SHIME^®^), Šuligoj et al. [59] recently reported that fermentation of 2′-FL resulted in a simultaneous increase in *Bifidobacterium* and SCFAs including butyrate in particular. In our *in vitro* experiments, fermentation of stool samples with 2′-FL also resulted in a simultaneous increase in *Bifidobacterium* and SCFAs including butyrate. As expected, the highest contribution to the SCFA increase was due to the production of acetate probably through the growth of *Bifidobacterium* spp., but significant increments of propionate and butyrate also occurred. Overall, levels of acetate, propionate and butyrate in the presence of 2′-FL were similar in the three stool donor groups after 24 h fermentation, indicating that 2′-FL could be applicable for both IBS and IBD. No major differences were observed between 2′-FL alone and the 2′-FL/probiotic combinations on the stool microbiota composition after 24 h of fermentation and some of the differences detected could be related to baseline variations. Because no additional effects were seen with the addition of the probiotic strains, we concluded that combining any of the tested probiotic strains with 2′-FL might not confer additional benefit to the host upon translation of the *in vitro* findings into human subjects. Hence we proceeded with including 2′-FL without probiotics in the nutritional formula tested in the pilot trial.

In the clinical trial, measures of gastrointestinal quality of life improved in adults with chronic gastrointestinal conditions after a six-week course of the 2′-FL-containing nutritional formula. Most notably, GIQLI total score and specifically the gastrointestinal symptom domain score improved significantly over the course of the trial. The increase in GIQLI total score (15.1 units) over six weeks exceeded what has been reported as the minimal clinically important difference (MCID) for the GIQLI (6.42 to 7.64 units) [60]. Improving gastrointestinal quality of life is meaningful given the negative impact of IBS, IBD, and celiac disease on health-related quality of life [61,62,63].

Over the course of the trial, stool microbiota counts increased at the genus and species levels. Consistent with our *in vitro* fermentations and other *in vitro* studies [35,64,65,66,67], the genus *Bifidobacterium* increased as expected. The increase in stool counts of *Bifidobacterium*, after consuming four grams 2′-FL per day for six weeks, is also in alignment with results demonstrating that two weeks of supplementation with ten grams per day of 2′-FL [41] or four weeks of supplementation with a ten gram mixture of 2′-FL and lacto-N-neotetraose [42] increased stool abundance of the genus *Bifidobacterium* in healthy adults and adults with IBS, respectively.

At the species level, *Bifidobacterium longum* also increased over time. This finding was also anticipated, because *B. longum* strains produce enzymes and transporters that function in the metabolism of HMOs and several *in vitro* studies have demonstrated that specific strains of *B. longum* are able to consume 2′-FL in culture [33,64,65,66,67]. This finding was also consistent with those of Elison et al. [36] showing that the increase in sequence abundance of operational taxonomic units (OTUs) showing high sequence similarity to *B. longum* were among the species of bifidobacteria most affected by 2′-FL consumption in human subjects.

Stool counts of *Faecalibacterium prausnitzii*, *Anaerotruncus colihominis*, and *Pseudoflavonifractor* species also increased significantly over the course of the trial. The increase in *F. prausnitzii* is particularly noteworthy because multiple studies have found that stool levels of *F. prausnitzii*, which comprises about 5% of total stool microbiota in healthy individuals, are significantly lower in patients with IBS, IBD, and celiac disease [9,68,69,70,71,72]. Due to the association between low abundance of *F. prausnitzii* and inflammatory and metabolic diseases, *F. prausnitzii* has been described as a potential bioindicator of health [73,74]. Furthermore, *F. prausnitzii* has been described as a potential keystone species [75,76] signifying that it may be critical for maintaining the organization and diversity of the gut ecosystem through biotic interactions with other species [77]. Although changes in *F. prausnitzii* were not noted in our *in vitro* experiments, an increase in *Eubacterium rectale*/*Clostridium coocoides*, other main butyrate producer group, was detected. Interestingly, Cheng et al. [78] recently reported that when *B. longum* subsp. *infantis* and *F. prausnitzii* are co-cultured in the presence of 2′-FL, these bacteria grow faster in co-culture than in monocultures in the presence of 2′-FL. The *in vitro* results reported by Cheng et al. [78] are in alignment with our observed simultaneous increase in the species *B. longum* and the butyrate producer *F. prausnitzii* in human subjects. Furthermore, our previous work and that of other groups have also demonstrated that prebiotics can simultaneously increase stool counts of *Bifidobacterium* and *F. prausnitzii* in adult subjects [79,80].

Stool levels of butyrate, acetate, and total SCFAs also increased significantly over the course of the clinical trial. Our *in vitro* experiments as well as other pre-clinical data also support these clinical trial findings. It was reported that incubation of infant stool samples with 2′-FL resulted in significantly increased SCFA production [81]. In several *in vitro* experiments, Li et al. [82] demonstrated that incubation of crude HMOs isolated from pooled human milk samples with stool samples isolated from piglets resulted in SCFA production. Furthermore, Azagra-Boronat et al. [83] recently reported that oral 2′-FL increased cecal butyrate in healthy suckling rats.

It is possible that the observed increases in SCFAs in the clinical trial, specifically butyrate and acetate, were mechanistically related to the observed increases in commensal bacteria. Stool levels of predominant butyrate producers have been shown to correlate with stool SCFA levels [84]. Microbiota of the genera *Faecalibacterium*, *Anaerotruncus*, *Pseudoflavonifractor*, each express enzymes required for butyrate and acetate biosynthesis [85]. *A. colihominis* is also considered a major butyrate producer [86,87]. Furthermore, specific strains of *B. longum* have been shown to produce abundant acetate when cultured on 2′-FL as a growth substrate [64,65]. Potentially supporting the growth of butyrate-producing gut microorganisms as well as intestinal production of butyrate may be clinically meaningful, since it has been suggested that exogenous butyrate and butyrate-producing microorganisms, such as *F. prausnitzii*, may represent novel therapeutic approaches for IBS and IBD [13,88,89,90,91,92].

Strengths of the translational clinical trial design include successful collection of pilot data in individuals with various gastrointestinal conditions and concurrent evaluation of impact on quality of life, gut microbiota, and stool SCFAs following *in vitro* assessments. Limitations of the clinical trial include a high rate of withdrawal, lack of a control group, the small sample size, and completion of the trial by only one individual with celiac disease. Furthermore, although participants who completed the trial were considered normal or overweight (but not obese), protocol eligibility criteria allowed for inclusion of a broad BMI range; future trial protocols should limit BMI to fewer categories and/or include sufficient sample size to allow for subgroup comparisons due to the influence BMI could have on results. In addition, shifts in gut microbiota composition and increases in SCFA cannot be attributed to 2′-FL solely given that the formula contained additional ingredients. Improvements in intra- and extra-intestinal symptoms also cannot be attributed only to 2′-FL given that the formula also contained macro- and micronutrients that are supportive to patients with malabsorption and malnutrition associated with chronic gastrointestinal disease including IBD [93,94].

## 5. Conclusions

Consistent with documented effects of 2′-FL, our *in vitro* work demonstrated bifidogenic and butyrogenic effects of 2′-FL during fermentation with human stool samples. With no additional effects seen with the addition of probiotic strains, only 2′-FL was included in the nutritional formula when the *in vitro* results were translated to human subjects. Consumption of the 2′-FL-containing nutritional formula by adults with IBS or ulcerative colitis was associated with improvements in intra- and extra-intestinal symptoms, and bifidogenic and butyrogenic effects. Based on the promising yet preliminary clinical trial results, future research should further explore relationships between potential impacts of the nutritional formula on quality of life and gut microbiota composition and metabolism, including SCFA production. To better isolate the impact of 2′-FL, the effect of the 2′-FL-containing formula could be compared to 2′-FL only or the formula without the inclusion of 2′-FL. Patient populations tested should include individuals with gastrointestinal conditions associated with intestinal dysbiosis, including, but not limited to IBS, IBD, and celiac disease. In addition, impact on the prevention of these conditions could be explored. Furthermore, additional batch culture fermentation experiments could explore effects of 2′-FL during fermentation with human stool samples from additional clinical populations, including those with other gastrointestinal conditions. However, the present results are promising, particularly in the context of the limited research on HMOs in adult populations with chronic gastrointestinal conditions.

## Figures and Tables

**Table 1:** Potential Synbiotic Combinations Evaluated *In Vitro.*

Synbiotic 1 (S1)	2′-FL + *Lactobacillus acidophilus* NCFM (ATCC 5221) + *Bifidobacterium animalis* subsp. *lactis* Bi-07 (ATCC 5220)
Synbiotic 2 (S2)	2′-FL + *L. acidophilus* NCFM + *B. animalis* subsp. *lactis* Bi-07 + *Bifidobacterium animalis* subsp. *lactis* Bl-04 (ATCC 5219) + *Lactobacillus paracasei* Lpc-37 (ATCC 5275)
Synbiotic 3 (S3)	2′-FL + *L. acidophilus* NCFM + *B. animalis* subsp. *lactis* Bi-07 + *Lactobacillus rhamnosus* GG (ATCC 7017)
Synbiotic 4 (S4)	2′-FL + *L. rhamnosus* GG
Synbiotic 5 (S5)	2′-FL + *Lactobacillus salivarius* UCC118

**Table 3:** Pilot Trial Participant Demographics.

	Mean ± SD or n (%)
Age (years)	31.4 ± 10.5
Sex	
Male	7 (58%)
Female	5 (42%)
Body Mass Index (kg/m^2^)	23.8 ± 3.4
Race (Ethnicity)	
White (not Hispanic/Latino)	8 (66.7%)
White/Native American (not Hispanic/Latino)	2 (16.7%)
White (Hispanic/Latino)	1 (8.3%)
Black (not Hispanic/Latino)	1 (8.3%)
Gastrointestinal Condition	
Irritable bowel syndrome (IBS)	7 (58.3%)
Ulcerative colitis (UC)	4 (33.3%)
Celiac disease	1 (8.3%)

Abbreviations: kg/m^2^, kilograms per square meter; SD, standard deviation.

**Table 4:** Pilot Trial Gastrointestinal Quality of Life Questionnaire Score Changes Over 6 Weeks.

	Score Range	Baseline		Week 6		Δ Over 6 Weeks	Mean % Δ	*p* ^1^	Cohen’s D
		Mean	SD	Mean	SD				
GIQLI Total Score	0–144	94.3	25.5	109.4	19.2	15.1	20.8%	0.020	0.59
GIQLI-Gastrointestinal Symptoms	0–76	53.3	10.3	61.4	7.7	8.1	18.1%	0.022	0.79
GIQLI-Physical Function	0–28	15.6	7.4	17.8	6.1	2.2	36.5%	0.164	0.30
GIQLI-Social Function	0–16	10.7	3.8	12.3	3.7	1.6	18.4%	0.004	0.42
GIQLI-Emotional Function	0–20	12.0	5.8	14.7	4.5	2.7	46.5%	0.139	0.47
IBDQ Total Score	32–224	134.0	52.4	179.0	36.1	45.0	43.6%	0.078	0.86
IBDQ-Bowel Symptoms	10–70	43.0	19.4	56.3	11.7	13.3	44.3%	0.165	0.69
IBDQ-Systemic Systems	5–35	16.8	6.2	27.0	6.3	10.2	66.9%	0.000	1.65
IBDQ-Social Function	5–35	22.0	9.9	27.8	7.5	5.8	41.5%	0.239	0.59
IBDQ-Emotion Health	12–84	51.5	18.7	68.0	11.7	16.5	43.3%	0.098	0.88
DSFQ Total Score	0–16	9.0	3.8	8.4	5.0	−0.6	−10.3%	0.522	−0.16

^1^*p*-values calculated using paired *t*-tests. Abbreviations: Δ, change; DSFQ, Digestive Symptom Frequency Questionnaire; GIQLI, Gastrointestinal Quality of Life Index; IBDQ, Inflammatory Bowel Disease Questionnaire; SD, standard deviation.

**Table 5:** Pilot Trial Stool Bacteria Count Changes Over 6 Weeks.

Phylum	Microbiota (CFU/g Stool)	Log Transformed Data						
		Baseline		Week 6		Geometric Mean % Δ	*p* ^1^	Cohen’s D
		Mean	SD	Mean	SD			
Actinobacteria	*Bifidobacterium* spp.	20.1	1.6	21.6	1.7	356.3%	* 0.026	0.95
	*Bifidobacterium longum*	17.4	2.0	19.4	1.6	650.0%	* 0.003	0.99
	*Collinsella aerofaciens*	17.6	3.0	18.1	3.5	65.7%	0.392	0.17
Bacteroidetes	*Bacteroides-Prevotella* group	20.2	0.9	21.2	1.3	169.0%	0.101	1.09
	*Bacteroides vulgatus*	19.8	2.5	20.9	2.6	190.4%	0.081	0.43
	*Barnesiella* spp.	16.0	2.0	15.4	2.9	−46.1%	0.468	−0.31
	*Odoribacter* spp.	15.6	2.5	14.8	3.6	−54.9%	0.369	−0.32
	*Prevotella* spp.	14.1	2.0	14.9	1.8	116.2%	0.301	0.39
Euryarchaeota	*Methanobrevibacter smithii*	13.1	2.0	14.0	2.1	134.7%	0.290	0.42
Firmicutes	*Anaerotruncus colihominis*	14.5	1.6	16.1	1.1	402.6%	* 0.018	1.00
	*Butyrivibrio crossotus*	9.9	1.5	10.7	3.1	111.7%	0.422	0.51
	*Clostridium* spp.	21.0	1.6	21.9	1.9	156.9%	0.125	0.58
	*Coprococcus eutactus*	14.1	2.3	14.1	2.7	−2.4%	0.983	−0.01
	*Faecalibacterium prausnitzii*	20.6	2.2	22.1	1.8	353.7%	* 0.029	0.67
	*Lactobacillus* spp.	19.5	2.5	20.5	2.4	160.7%	0.252	0.39
	*Pseudoflavonifractor* spp.	18.0	1.5	19.2	1.2	226.4%	* 0.016	0.80
	*Roseburia* spp.	20.7	1.8	21.9	1.5	252.8%	0.062	0.68
	*Ruminococcus* spp.	17.7	2.0	18.4	2.1	97.5%	0.273	0.34
	*Veillonella* spp.	16.1	3.0	16.9	1.7	118.8%	0.331	0.26
Fusobacteria	*Fusobacterium* spp.	9.6	2.3	10.6	2.0	171.8%	0.111	0.44
Proteobacteria	*Desulfovibrio piger*	10.3	2.1	10.5	2.0	12.2%	0.877	0.06
	*Escherichia coli*	16.5	3.0	17.0	1.7	67.9%	0.607	0.17
	*Oxalobacter formigenes*	14.1	1.7	13.9	2.5	−16.9%	0.833	−0.11
Verrucomicrobia	*Akkermansia muciniphila*	12.6	2.0	12.9	2.0	33.6%	0.702	0.15

^1^*p*-values calculated using paired *t*-tests. * *p*-values confirmed with a Wilcoxon signed rank test (*p* < 0.05). Abbreviations: Δ, change; CFU, colony-forming unit; g, gram; SD, standard deviation; spp., species.

**Table 6:** Pilot Trial Stool Short-Chain Fatty Acids (SCFAs) Over 6 Weeks.

SCFA (Micromol/g Stool)	Baseline		Week 6		Mean % Δ	*p* ^1^	Cohen’s D
	Mean	SD	Mean	SD			
Total SCFA	46.30	13.30	76.43	37.04	72.2%	0.026	2.27
Butyrate	8.12	4.76	16.71	9.63	594.0%	0.040	1.80
Acetate	28.24	7.28	45.65	23.90	64.3%	0.035	2.39
Propionate	10.03	4.71	14.06	8.39	42.4%	0.074	0.85

^1^*p*-values calculated using paired *t*-tests. Abbreviations: Δ, change; g, gram.

## Data Availability

Data is contained within the article and supplementary material.

## Supplementary Materials

The following are available online at https://www.mdpi.com/2072-6643/13/3/938/s1, Figure S1: Bacterial populations at 0 h, 8 h, and 24 h of fermentation with no substrate/negative control (NC), positive control (FOS/inulin mixture) and the prebiotic 2′FL.
