# Supplementary material for: Impact of 2′-Fucosyllactose on Gut Microbiota Composition in Adults with Chronic Gastrointestinal Conditions: Batch Culture Fermentation Model and Pilot Clinical Trial Findings
Source: Nutrients. 2021 Mar 14;13(3):938. doi: 10.3390/nu13030938 (PMC7998190; doi:10.3390/nu13030938)
Supplement: Supplementary file 1 [file nutrients-13-00938-s001.pdf]

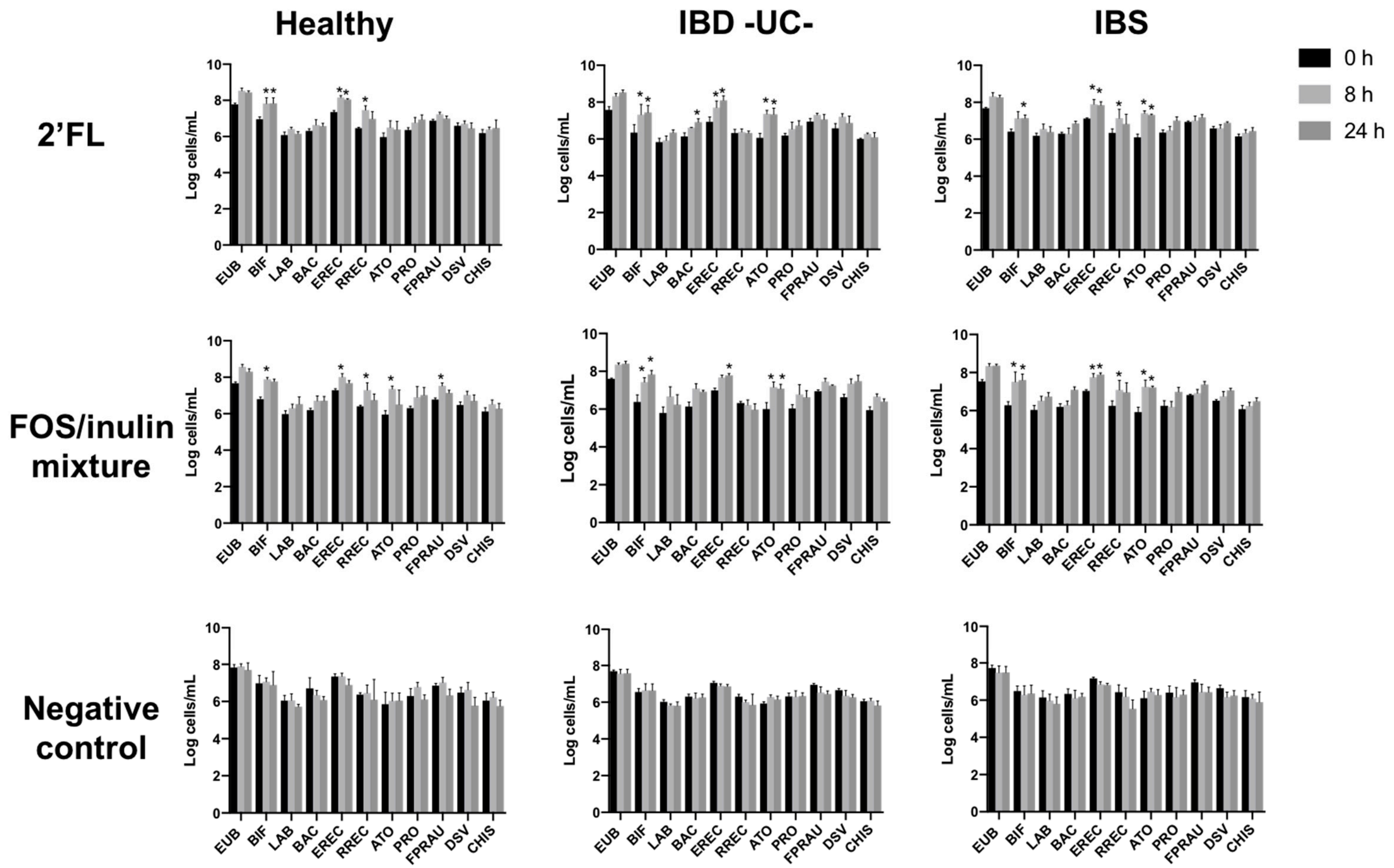

Figure S1: Bacterial populations at 0 h, 8 h, and 24 h of fermentation with no substrate/negative control (**NC**), positive control (**FOS/**inulin mixture) and the prebiotic **2'FL**. Error bars show SEM (n=3). Significant differences from baseline (time 0) are denoted with*(p ≤ 0.05
Eub -total bacteria-; Bif -*Bifidobacterium*-; Bac - *Bacteroides-Prevotella*-; Lab - *Lactobacillus-Enterococcus-;* Ato - *Atopobium* cluster-; Prop -propionate producing bacteria (Clostridium cluster IX)-; Erec - *Eubacterium rectale-Clostridium cocoides* group (*Clostridium* cluster XIVa and XIVb)-; Rrec- *Roseburia* spp -; Fprau - *Faecalibacterium prausnitzii* cluster -; Chis- *Clostridium histolyticum* group (*Clostridium* cluster I and II)-; DSV- *Desulfovibrionales* -.
